# Comparative repeatome analysis of Pyrgomorphidae and Acrididae (Orthoptera: Caelifera) revealed the contribution of repetitive DNA in genome gigantism

**DOI:** 10.1371/journal.pone.0325165

**Published:** 2025-06-02

**Authors:** Muhammad Salman, Xuanzeng Liu, Nian Liu, Yuan Huang

**Affiliations:** College of Life Sciences, Shaanxi Normal University, Xi’an, China; Quinnipiac University, UNITED STATES OF AMERICA

## Abstract

Eukaryotic genomes are often rich in DNA repetitive elements, involving both transposable elements (TE) and tandemly repeated satellite DNA. Grasshopper species, known for their large genome sizes, comprising relatively a high proportion of genomic repeats. This study aimed to identify and perform a comparative analysis of DNA repetitive content in eight grasshopper species from the Pyrgomorphidae and Acrididae families. We utilized unassembled low-coverage Illumina paired-end short reads in the RepeatExplorer2 pipeline to identify genomic repeats, and RepeatMasker to estimate their abundance and divergence activity. Flow cytometry estimated genome sizes, ranging from 1C = 7.670 pg to 18.612 pg, with *Aularches miliaris* (18.612 pg) being the second largest insect genome reported to date. The repeat content ranged from 51% to 74%, with a mean value of 64.26% of the total genome. The major identified repeat elements included LINE, Ty3_Gypsy, Penelope, Ty1-copia, Helitron, Maverick, and satellite repeats, with LINE elements being the most abundant, constituting 24% to 54% of the total repetitive content in *Apalacris varicornis* and *A. miliaris*, respectively. The positive correlation of repetitive content and TEs with genome size suggests that their expansion has contributed to the large genome sizes observed. Satellite DNA analysis identified 65 satDNA families across the eight species. Additionally, phylogenetic analysis of TE protein domains revealed that consensus sequences from the same domain cluster together, suggesting domain-specific evolutionary pathways for TEs in the genome. This study reveals new dynamics into the role of repetitive DNA in genome gigantism as well as other evolutionary mechanisms in the Pyrgomorphidae and Acrididae families of Orthoptera.

## Introduction

Repetitive DNA elements constitute a significant portion of eukaryotic genomes and play a crucial role in driving genomic evolution in host organisms [1]. These repeat sequences are classified into two major classes: tandemly repeated satellite DNA (satDNA) and interspersed transposable elements (TEs), collectively termed as “repeatome” [2]. Tandem repeats include satDNA, a non-coding sequence that is repeated multiple times, primarily in the heterochromatin regions of centromeres and pericentromeres, as well as in subtelomeric and interstitial regions in most eukaryotic genomes [3,4]. Based on nucleotide sequences, satDNA is further classified into microsatellites, minisatellites, and satellites [5,6]. Previously, satellite DNAs were thought to be non-functional or junk DNA, but recent studies have suggested various functional roles. The most widely recognized function involves centromere activity, including kinetochore assembly, meiotic chromosome segregation, X chromosome recognition, and heterochromatin formation via the siRNA pathway [7–9]. Additionally, recent research has shown that satDNA transcription contributes to cellular stress responses, environmental adaptation, gene expression regulation, and cancer progression [4,10–12]. With an increasing understanding of these particular sequences, the term “satellitome” was recently introduced to describe the entire set of satDNA in a genome [13].

Transposable elements are another important type of widely distributed repetitive elements in eukaryotic genome [14]. TEs are categorized into two classes based on their transposition mechanisms: Class I retrotransposons and Class II DNA transposons. Class I elements replicate through a “copy and paste” mechanism involving an RNA intermediate, whereas Class II elements utilize a “cut and paste” process of transposition that does not require an RNA intermediary. Class I retrotransposons include long terminal repeats (LTRs), short interspersed nuclear elements (SINEs), and long interspersed nuclear elements (LINEs). In contrast, Class II DNA transposons encompass elements such as Maverick, Helitron, Crypton, and terminal inverted repeats (TIRs) [15–17].

Genome sizes vary greatly within the Tree of Life. Eukaryotic species exhibit a remarkable 200,000-fold variation, and within the Animal kingdom, there is a 7,000-fold difference in haploid genome sizes (C-value) [18,19], ranging from 0.02 to 132.83 pg [20]. Orthoptera is the only group of insects that exhibit an expanded genome size, with a 20-fold variation. The smallest recently measured genome is 0.95 pg from *Oecanthus euryelytra* Ichikawa (Ensifera, Gryllidae) [21], and the largest is 21.96 pg from *Bryodemella tuberculata* Fabricius (Caelifera: Acrididae) [22]. It has been remained the fascinating subject for the scientists to uncover the evolutionary dynamics behind these gigantic genomes. Some researchers have proposed that larger genomes in certain species, such as the salamanders [23] and mountain grasshopper *Podisma pedestris* [24], could be due to lower DNA loss from the genome. This phenomenon stands in contrast with the mammals and birds, which generally have smaller genome sizes [23,25]. While other studies have suggested that larger genomes in grasshoppers might be the effect of whole genome duplication (WGD) or due to the repetitive DNA expansion, mainly TEs, satDNAs and also could be the lower rate of piRNA silencing in the host genome [20,26–29].

Previously, there was very poor and less descriptive information available related to repetitive DNA especially for the satellite repeats. However, with the advent of next-generation sequencing (NGS) technologies, investigations into repetitive DNA have significantly improved [30]. Genome assembly processes have been transformed with NGS like Illumina PE sequencing [31], making it possible to study repetitive elements within highly complex genome sequences of plants and animals [32,33]. Repeatome investigations involving both transposable elements and satellite DNA were previously less understood in Orthoptera. However, recently, a few studies have been reported for *Vandiemenella viatica* [34], *Locusta migratoria* [13], *Angaracris rhodopa* [20], *Oedaleus decorus* [35], the genus *Calliptamus* [29], the suborder Gryllidea [19] and the families Acrididae [36] and Pamphagidae [37].

The family Acrididae (Orthoptera: Caelifera: Acridoidea) is a major diversified group of insects, with almost 6,700 known species found worldwide [38]. These grasshoppers are notorious pests, known for their destructive impact on both food and cash crops [39]. The grasshopper family Pyrgomorphidae (Orthoptera: Caelifera: Pyrgomorphoidea), renowned for its vibrant colors, includes approximately 500 species globally. They are often called gaudy or bush grasshoppers, several of these species sequester plant secondary compounds. Many are significant agricultural pests, while others hold cultural importance [40]. The current study involves the selection of unassembled Illumina paired-end short reads of eight species from the two families mentioned above to identify and compare the repeat elements of their genomes using the RepeatExplorer2 clustering pipeline and RepeatMasker. This comparative investigation offers strong foundation into the evolutionary dynamics of TEs and satDNA in genome size variation, providing significant implications for future genomic studies of the order Orthoptera.

## Materials and methods

### Sample collection, genome size estimation and next-generation sequencing

The selected eight species from two families (Pyrgomorphidae and Acrididae) were collected from natural populations in various locations of Yunnan Province, China, geographical details of each sample are specified in supplementary material (S1 Table in S2 File). The collected samples were stored at −80°C for further genome size estimation and sequencing at the laboratory of molecular evolutionary biology, College of Life Science, Shaanxi Normal University, Xi’an, China.

We have used flow cytometry (FCM) of propidium iodide-stained nuclei to estimate the genome sizes and utilized the head tissues from the freshly collected samples while following the standard protocols. The coefficient of variation (CV) was ensured to be less than 5% for all 2C peaks and under each 2C peak at least 10,000 cells were measured. This was performed using a CyAn ADP flow cytometer at the 488 nm laser tuning (Beckman Coulter, Indianapolis, IN, USA). We compared the ratio of mean 2C of standard to that of mean 2C of sample to estimate DNA content [41]. The Male *L. migratoria* was used as internal standard in this experiment. Genome size in base pairs (bp) was converted using the formula: genome size (bp) = (0.978 × 10^9) × DNA content (pg), which utilizes the conversion factor accurately [42,43].

The genomic DNA was extracted utilizing the Genomic DNA Kit, TIANamp (Tiangen Biotech, Beijing, China) by following the instructions of the manufacturer. The DNA extracted was then sonicated to obtain fragments approximately 350 bp in length and the libraries were prepared and fixed onto a microarray by bridge PCR. The next generation sequencing (NGS) was performed using Illumina NovaSeq 6000 (PE150 bp) platform.

### Read sampling, quality control and pre-processing of reads

The unassembled illumina paired-end reads, generated from a total sequencing dataset of ~4G bases per species (S1 Table in S2 File), were used in the RepeatExplorer2 (RE2) pipeline for repetitive DNA analysis, which requires a genome coverage of 0.1–0.5× for optimal performance [44,45]. To achieve this, we utilized SeqTK (https://github.com/lh3/seqtk, last accessed May 2024) to randomly sample 6,000,000 reads from each read file at the command line (seqtk sample -s 10 input_file 6000000 | gzip -c > output_sample_file). Random sampling ensured that the selected reads were representative of the entire genome. The sampled files for all selected species were then uploaded to the RepeatExplorer2 Galaxy server using the FTP upload option (curl -T output_sample.file -k -v -u username ftps://repeatexplorer-elixir.cerit-sc.cz).

The read quality checks were conducted using FastQC, available within the RepeatExplorer2 (RE2) Galaxy server tools (last accessed May 2024) and read preprocessing was performed using the “Preprocessing of FASTQ Paired-End Reads” tool (last accessed 16 May 2024) with default settings. This preprocessing step included adapter detection, quality filtering, read trimming, cut-adapt filtering, removal of single-end reads, and interlacing of paired-end reads into a single FASTQ file.

### Individual clustering analysis for repeat characterization

Individual clustering analysis was performed to identify and annotate repeat content in each species separately. The RepeatExplorer2 (RE2) tool (https://repeatexplorer-elixir.cerit-sc.cz/galaxy/, last accessed May 2024) utilized the interlaced paired-end reads file from the preprocessing step as the input. The options were set to “Select queue” as “long” and the database as “Metazoa version 3.0,” following the procedures described by the developers [46]. The RE2 pipeline generated three output files: an HTML report, a log file, and an archive containing the HTML report. These files were downloaded for inspection, manual curation, and annotation of repeats that were unannotated by the RE2 pipeline. To validate RepeatExplorer2 results and detect potential contamination from foreign DNA, BLASTn searches (https://blast.ncbi.nlm.nih.gov/Blast.cgi, last accessed March 2025) were performed on rank 1–4 TAREAN consensus sequences from the RepeatExplorer2 output archive against the NCBI nucleotide (nt) database. Additionally, RepeatProfiler v1.1 (https://github.com/johnssproul/RepeatProfiler, last accessed March 2025) was used to map repeat consensus sequences to raw NGS reads and generate repeat profiles. FASTA-formatted consensus sequences were mapped against 6 million randomly sampled reads from each species to assess repeat profiles and coverage distributions.

### Comparative clustering analysis

In the comparative analysis, read names were assigned a three-letter species-specific code to the paired-end reads file generated during the preprocessing step, using the “FASTA Read Name Affixer” tool (last accessed May 2024) on the RE2 platform. The species-specific code was derived by taking the first letter of the genus name and the first two letters of the species name, formatted as a three-letter capitalized prefix (e.g., the species code for *Atractomorpha yunnanensis* is AYU, as shown in S3 Table in S2 File). Subsequently, the subsampling of reads was performed using the “Read Sampling” tool, reducing the reads to 500,000 per species. The datasets from all 8 species were then concatenated into a single file using the “Concatenate Datasets” option, resulting in a single FASTA file containing 4,000,000 reads.

The concatenated FASTA file was used as the input dataset for comparative clustering analysis with the RepeatExplorer2 pipeline (https://repeatexplorer-elixir.cerit-sc.cz, last accessed May 2024). The pipeline was run in comparative mode with the following parameters: paired-end reads set to “yes,” REXdb set to “Metazoa version 3.0,” advanced options enabled, perform comparative analysis set to “yes,” group code length set to “3,” and queue selection set to “long,” while all other options were kept as default. The output files generated from the clustering analysis were downloaded and manually inspected for possible annotations. The results of the comparative clustering analysis were visualized using the “plot_comparative_clustering_summary.R” script available under “Visualization of Comparative Clustering” in RepeatExplorerUtilities (last accessed May 2024). Two output files from the RE2 archive, CLUSTER_table.csv and COMPARATIVE_ANALYSIS_COUNTS.csv, were utilized for generating the visualizations.

### TEs genetic divergence and expansion activity

The genetic divergence of transposable elements was analyzed using RepeatMasker v4.1.6 (https://repeatmasker.org/, last accessed, December 2024) to estimate their insertion and expansion activity across the genomes. For this analysis, we employed Orthoptera-TElib [47], a specialized library for TE annotation in Orthoptera insects, by utilizing the -lib option and the “RMBlast” engine in RepeatMasker. Additionally, the “calcDivergenceFromAlign.pl” script was used to estimate divergence values, and the “createRepeatLandscape.pl” script was applied to generate Kimura divergence landscapes.

### Comparative Satellitome analysis and divergence of satellite repeats

The RE2 pipeline includes the Tandem Repeat Analyzer (TAREAN) tool, which was utilized for the identification of satellite repeats. Satellite DNA consensus sequences were extracted from the comparative clustering analysis, and a standardized nomenclature was followed to assign specific names to each satDNA family. The naming convention involved the first letter of the genus and the first two letters of the species name, followed by the word “Sat” and the specific monomer length of the satDNA family (e.g., ApsSat01–1212) [13]. All clusters identified by TAREAN were manually examined, and each consensus sequence was named according to the species contributing the majority of reads to that cluster. The script rm_homology.py from the satMiner toolkit (https://github.com/fjruizruano/satminer/blob/master/rm_homology.py/, last accessed December 2024) was employed to identify homologies and evaluate sequence similarities among satDNA families.

The genetic divergence and abundance of satDNA families were estimated using RepeatMasker v4.1.6 (https://repeatmasker.org/, last accessed December 2024) with the -a option and the -lib option for a customized library of consensus satDNA sequences. The script calcDivergenceFromAlign.pl was then used to calculate satDNA divergence. Finally, the createRepeatLandscape.pl script from the RepeatMasker suite was employed to generate satellitome landscapes for each species.

### Extraction of TE protein domain consensus sequences

Domain-based annotation of transposable elements (DANTE) under the RE2 pipeline was used to extract protein domain-based consensus sequences of TEs. In the first step, the “RepeatExplorer2 comparative clustering archive” file was utilized to extract contigs using the “Extract contigs from RepeatExplorer2 archive” option. The resulting contig FASTA file was then processed in the DANTE tool (last accessed 28 May 2024) with the following options: database set to ‘Metazoa_v3.1,’ iterative searches set to ‘No,’ and scoring matrix set to ‘BLOSUM80.’ Finally, consensus sequences of TEs were generated using the “Extract Domains Nucleotide Sequences” tool available in DANTE. Individual sequences in the final FASTA file were renamed using the first letter of the repetitive element and the first two letters of the specific protein domain (e.g., LRT for LINE elements with reverse transcriptase-RT) for subsequent use in phylogenetic analysis.

### Phylogenetic analysis

We conducted two distinct phylogenetic analyses to investigate the evolutionary relationships among the genomes studied. The first analysis was based on 15 mitochondrial sequences, including 13 protein-coding genes (PCGs) and 2 rRNA, to infer genome-wide phylogeny. Mitochondrial sequences were extracted using MitoFinder v1.4.2 (https://github.com/RemiAllio/MitoFinder), with *Locusta migratoria* mitochondrial sequences in GenBank format (NCBI Accession No: NC_011119.1) serving as the reference database [28]. The second analysis focused on protein domain-based consensus sequences of TEs. For both analyses, multiple sequence alignments were performed using MAFFT v7.453 (https://mafft.cbrc.jp/alignment/software/), and phylogenetic tree construction was conducted using IQ-TREE v2.3.4 (http://www.iqtree.org/). IQ-TREE employed the combined options of best-fit model auto-selection via ModelFinder and ultrafast bootstrap with 1,000 replicates (iqtree -s alignment.phy -m MFP -B 1000). The resulting phylogenetic trees were visualized and edited using FigTree v1.4.4.

## Results

### Genome size, repeat proportions and composition

**Fig 1:**
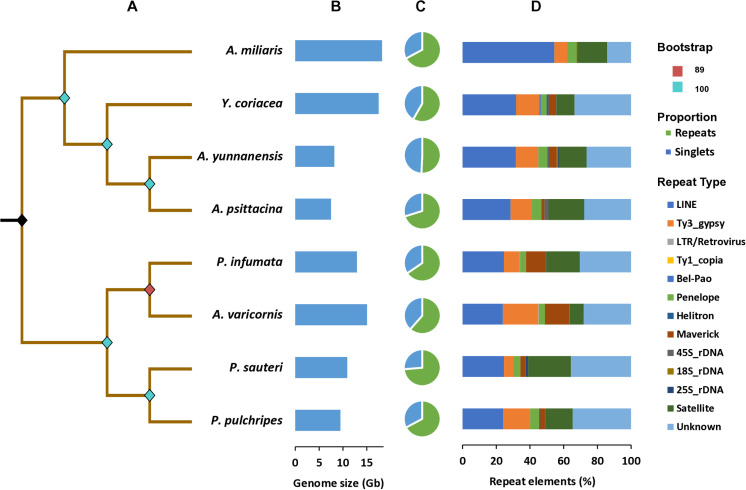
Genome repeat proportion and composition of DNA repetitive content Pyrgomorphidae and Acrididae. (A) Phylogenetic analysis based on 13 mitochondrial protein coding genes (PCGs) and 2 rRNA genes, the initial split into two clades separating both families with Pyrgomorphidae species on upper and Acrididae on lower branch. The color pattern indicates the bootstrap support. (B) Genome size across the species in Gb. (C) Singlets and repeats proportions across the species. (D) Repeat content composition of species with LINE, Ty3_gypsy and satellite repeats being abundant across the genomes.

Grasshoppers, a significant group of insects, typically exhibit large genome sizes, with some genomes being nearly six times larger than the human genome (3 Gb). The genome sizes were determined using flow cytometry (FCM). The results ranged from the smallest genome, 7.67 pg in *Atractomorpha psittacina*, to the largest genome, 18.612 pg in *A. miliaris*, both belonging to the Pyrgomorphidae family (Fig 1B and Table 1). Notably, in our study, *A. miliaris* was identified as the second-largest insect genome reported to date, surpassed only by *Bryodemella tuberculata* (21.96 pg) [22]. The Acrididae family also displayed considerably large genome sizes, with 15.378 pg in *A. varicornis*, followed by 13.220 pg in *Phlaeoba infumata*, 11.150 pg in *Pternoscirta sauteri* and 9.690 pg in *Pternoscirta pulchripes* (Fig 1B and Table 1).

**Table 1:** Species details and genome sizes of Pyrgomorphidae and Acrididae. The table includes the scientific names of the species, their family and superfamily classifications, and genome sizes presented in picograms (pg) and gigabases (Gb). Species classification is based on the Orthoptera Species File (last accessed December 25, 2024; https://orthoptera.speciesfile.org/).

Sr. No	Species Name	Super-family	Family	Genome size (Pg)	Genome size (Gb)
1	*Atractomorpha yunnanensis* (Bi & Xia, 1981)	Pyrgomorphoidea	Pyrgomorphidae	8.382	8.197
2	*Atractomorpha psittacina* (Haan, 1842)			7.670	7.501
3	*Aularches miliaris* (Linnaeus, 1758)			18.612	18.202
4	*Yunnanites coriacea* (Uvarov, 1925)			17.873	17.479
5	*Phlaeoba infumata* (Brunner von Wattenwyl, 1893)	Acridoidea	Acrididae	13.220	12.929
6	*Pternoscirta sauteri* (Karny, 1915)			11.150	10.904
7	*Apalacris varicornis* (Walker, 1870)			15.378	15.039
8	*Pternoscirta pulchripes* (Uvarov, 1925)			9.690	9.476

RE2 pipeline was utilized to perform the individual clustering analysis with the recommended 0.1–0.5 × genome coverage per sample. The genomic content of the eight selected species was found to be highly repetitive, with an average of 64.26% as repeats, ranging from 51% to 74% in *A. miliaris* and *A. varicornis*, respectively (Fig 1C). In total, the repetitive content revealed approximately 12 types of repeat elements, which included Class I retrotransposons LINE, LTRs, and Penelope, and Class II DNA transposons such as Helitron, Maverick, as well as tandem repeats like rDNA and satDNA (Fig 1D). The major LTRs found in the genomic repeat were Ty3_gypsy, Ty1_copia, Retrovirus and Bel-Pao, while main rDNAs were identified as 45S_rDNA, 25S_rDNA, and 18S_rDNA. The LINE element was consistent and the most abundant repeat across all the genomes, ranging from 24% to 54% of total repetitive content in *A. varicornis* and *A. miliaris*, respectively. This was followed by the retrotransposons Ty3_gypsy, which constituted 6% to 21%, and Penelope, which accounted for 3% to 6% in their respective species (Fig 1D). The DNA transposons, Helitron and Maverick, were also identified, having substantially lower percentages in most of the species. The elements, such as Ty1_copia, Retrovirus and Bel-Pao only accounted for a little fraction of genomic repeat content. Tandem Repeat Analysis (TAREAN) showed a significant proportion of satDNA in all eight species, with the highest at 25% (*P. sauteri*) and the lowest at 9% (*A. varicornis*) of total genomic repetitive content, while relatively a low fraction of rDNA was found across the species (Fig 1D).

**Fig 2:**
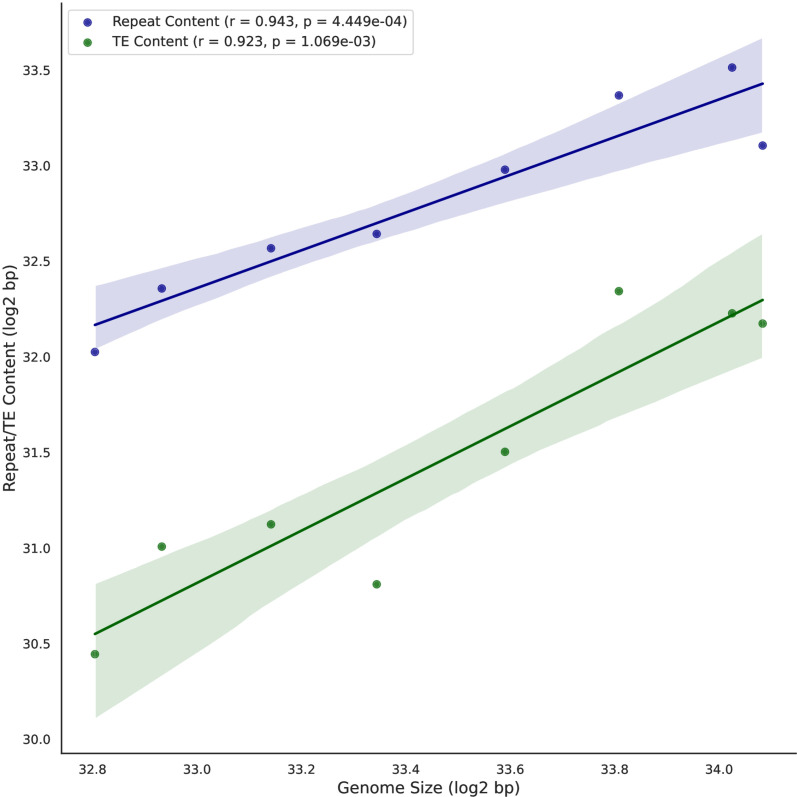
The correlation analysis between repeat content and TE content with genome size. Blue line shows strongly positive correlation between repeat content and genome size and green line indicates the strong positive correlation between TE content and genome size.

Additionally, we performed a correlation analysis to explore the relationship between repetitive content, TEs, and genome sizes. The analysis revealed a statistically significant and highly positive correlation between repetitive content and genome size (Pearson Correlation: r = 0.943, p = 4.449e-04) as well as between TE content and genome size (r = 0.923, p = 1.069e-03) (Fig 2). Likewise, the correlation analysis between genome size and major TE types, along with satDNA, revealed moderate to strong positive associations, except for rDNA, which is present in very low proportions within the genome (S2 Fig in S1 File). These findings suggest that the exceptionally large genomes observed in Orthoptera species, particularly in the families Pyrgomorphidae and Acrididae, may be driven by their highly repetitive genomic composition, especially the proliferation of TEs.

### Comparative analysis of repetitive elements

**Fig 3:**
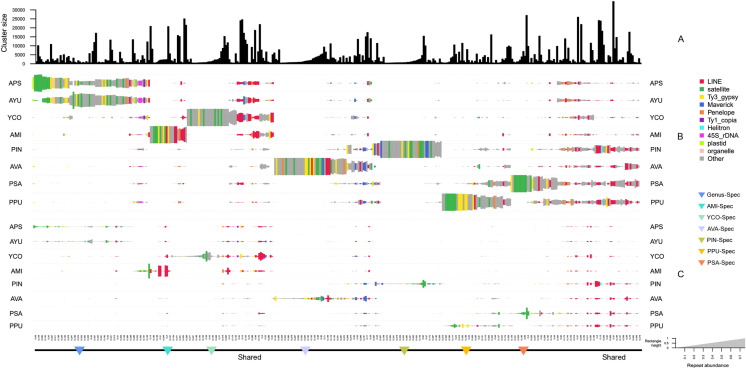
Comparative repeatome analysis visualization of Pyrgomorphidae and Acrididae. (A) The size of each cluster (number of reads) is represented in the top bar plot. (B) The scaled rectangles represent the proportion of individual repeats in each species. (C) The scaled rectangles are re-calculated based on genome size, representing the actual number of reads in the genome. The size of each peak shows the total read numbers in the respective cluster. The top clusters labeled as “shared” denote repeats that showed similar repeat landscape. “Genus-Spec” refers to repeats that are specific to a particular genus. “AMI/YCO/AVA/PIN/PPU/PSA-Spec” are sets of species-specific groups containing unique repeats.

We used the RE2 pipeline in comparative mode to investigate cross-species repeat content, resulting in a comparative analysis visualization (Fig 3) comprising three sections. The bar plot at the top represents each super-cluster, with the size of the bars indicating the total number of analyzed reads (Fig 3A). The scaled rectangles below the bar graph reveal the proportions of each repeat element in a particular species (Fig 3B). Most of the top clusters within the families Pyrgomorphidae and Acrididae exhibit similar repeat landscapes, likely due to a relatively shorter divergence time among these species (Figs 1A and 3B). In contrast, a distinct scale of divergence is observed between the two families, suggesting a comparatively higher evolutionary distance. The top clusters labeled as “shared” represent repeats that are similar in abundance among these species and typically include LINE, Ty3_gypsy, Helitron, Penelope, and some unclassified repetitive elements. Additionally, Ty1_copia and Maverick elements were also identified in these shared clusters. A fraction of clusters in each species has been categorized as species-specific, containing a unique set of repeats, except for *A. psittacina* and *A. yunnanensis*, which belong to the same genus and share a similar repeat pattern, and are therefore not indicated at the bottom line. Clusters labeled “AMI/YCO/AVA/PIN/PPU/PSA-Spec” predominantly contain satDNA, which is evolutionarily the most dynamic component of DNA repetitive elements (Fig 3). Furthermore, individuals from the Acrididae family also contain Ty3_gypsy and LINE elements, indicating the diversification and proliferation of these LTR and non-LTR retrotransposon lineages within their genomes.

Finally, the analyzed read numbers and total read counts within each cluster for the respective species were normalized relative to genome size (Gb). The height of the peaks in each cluster represents the actual number of reads corresponding to the respective repeats in the genome (Fig 3C). Normalization by genome size, particularly for the relatively larger clusters of LINE and satellite elements, revealed significantly taller peaks, indicating a higher number of reads within these clusters (Fig 3C).

### Evolutionary dynamics of TE divergence

**Fig 4:**
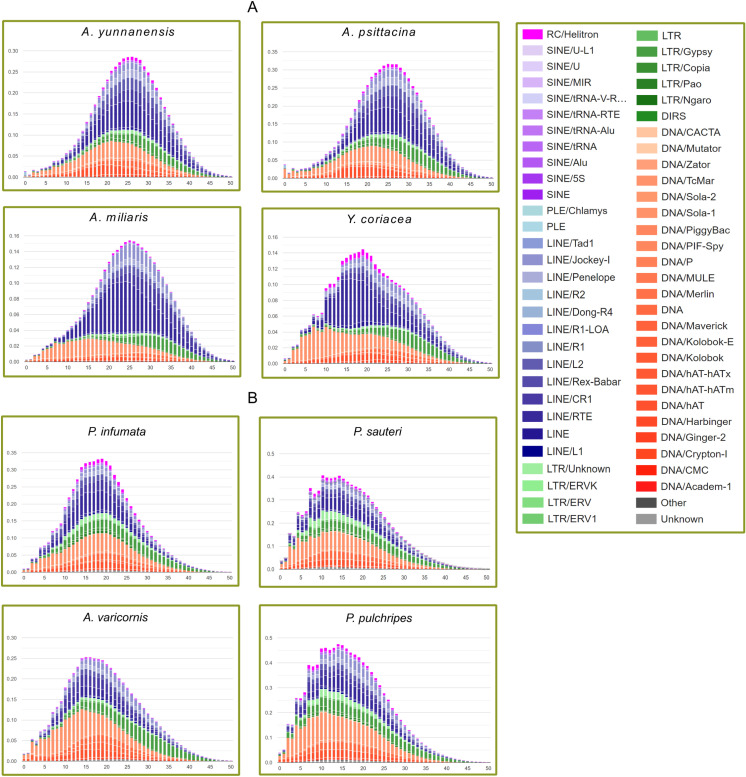
TE genetic divergence landscapes across the species. (A) TE divergence landscapes of family Pyrgomorphidae with x-axis indicating the Kimura divergence percentage and y-axis shows the proportion of genome occupied by each TE. (B) TE divergence landscapes of family Acrididae.

We used RepeatMasker v4.1.6 to estimate the divergence activity of transposable elements and to study the evolutionary dynamics of TE expansion across genomes. TE insertion patterns confirm whether TE integration into genomes reflects recent burst events or represents an ongoing process of accumulation and proliferation. The Kimura 2-parameter (K2P) distance from consensus sequences was employed to estimate TE accumulation times within genomes. TE divergence landscapes predominantly indicated recent insertion activity (K2P = 0–15) across the genomes (Fig 4). The family Pyrgomorphidae displayed a consistent bell-curve shaped repeat landscape, with relatively lower TE activity in recent times and showed evidence of ancient TE proliferation events in *A. yunnanensis*, *A. psittacina*, and *A. miliaris*, which might have contributed to their expanded genome sizes (Fig 4A). However, *Yunnanites coriacea* deviated from this pattern, exhibiting insertion peaks at both lower Kimura levels (0–5), indicating recent activity, and higher levels, reflecting older proliferation events. In contrast, species within the family Acrididae exhibited more recent TE activity (K2P = 0–10), particularly in *P. sauteri* and *P. pulchripes* (Fig 4B). The class I retrotransposons, including LINE and LTR elements, emerged as the most actively accumulating repeat components, followed by DNA transposons with comparatively lower proportions. These findings suggest that TE proliferation in these repeat lineages is still ongoing, driving the genome size expansion among these species.

### Comparative satellitome analysis

**Fig 5:**
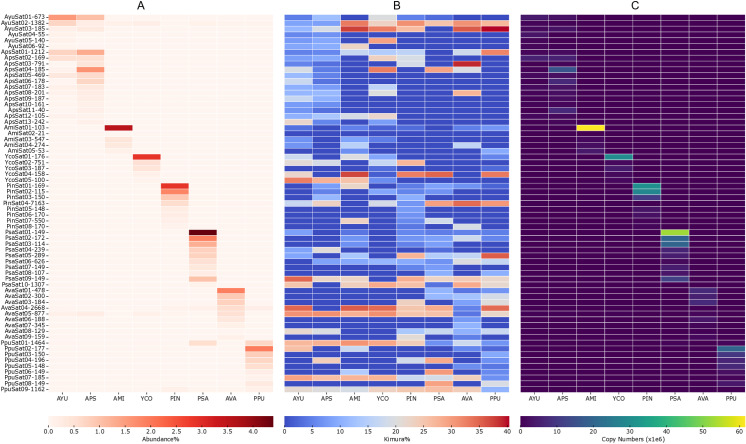
Heatmaps of percentage abundance, divergence and copy numbers of 65 satDNA families across the species. (A) The abundance percentage of 65 satDNA families across the genomes with PsaSat01 showing the highest proportion in *P. sauteri*.. (B) The divergence percentage satDNA families in the eight species. (C) The copy numbers of satDNA repeats occupied by each genome, represented in millions.

Comparative satellite DNA analysis using TAREAN in the RepeatExplorer2 pipeline identified 65 satDNA families across eight species of Pyrgomorphidae and Acrididae, with repeat unit lengths ranging from 21 nt in AmiSat02–7163 nt in PinSat04 (Fig 5). Among these, five satDNA families were consistently identified across the eight genomes. AmiSat02 was initially not mapped by RepeatMasker in any species, likely due to its extremely short monomer length (21 nt). However, when the sequence was extended by repeating the monomer to form dimers to pentamers, AmiSat02 was successfully mapped in *A. miliaris*, confirming its presence as a tandem repeat (S5 Table in S2 File). The satellite DNA consensus sequences were predominantly AT-rich, with A + T content ranging from 47.3% to 76.19% and a median of 59.27%. In contrast, the highest G + C content of 52.7% was observed in the PpuSat05 satDNA family.

Satellite repeat content varied significantly across the species, ranging from 4.44% in *Y. coriacea* to 12.21% in *P. sauteri* of the total genome (S4 Fig in S1 File). Species-specific satDNA families were abundantly mapped in their respective genomes, with PsaSat01 exhibiting the highest abundance at 4.39% of the total genomic content in *P. sauteri*, followed by AmiSat01 (3.54%) in *A. miliaris* and YcoSat01 (2.88%) in *Y. coriacea* (Fig 5A). These species-specific families displayed lower Kimura genetic divergence within their respective genomes and closely related species compared to distant species. For instance, AyuSat03 showed a divergence of 11.06% in *A. yunnanensis*, 19.02% in *A. psittacina*, and 40.35% in *P. pulchripes* (Fig 5B).

The copy numbers of satDNA families also varied widely, with AmiSat01 in *A. miliaris* showing the highest copy number of 61.61 million, followed by PsaSat01 in *P. sauteri* at 52.76 million (Fig 5C). SatDNA families that were more abundant in the genome also exhibited higher copy numbers, indicating that only a few satellite repeats are preferentially amplified, contributing to genome size expansion.

### Satellitome divergence activity

**Fig 6:**
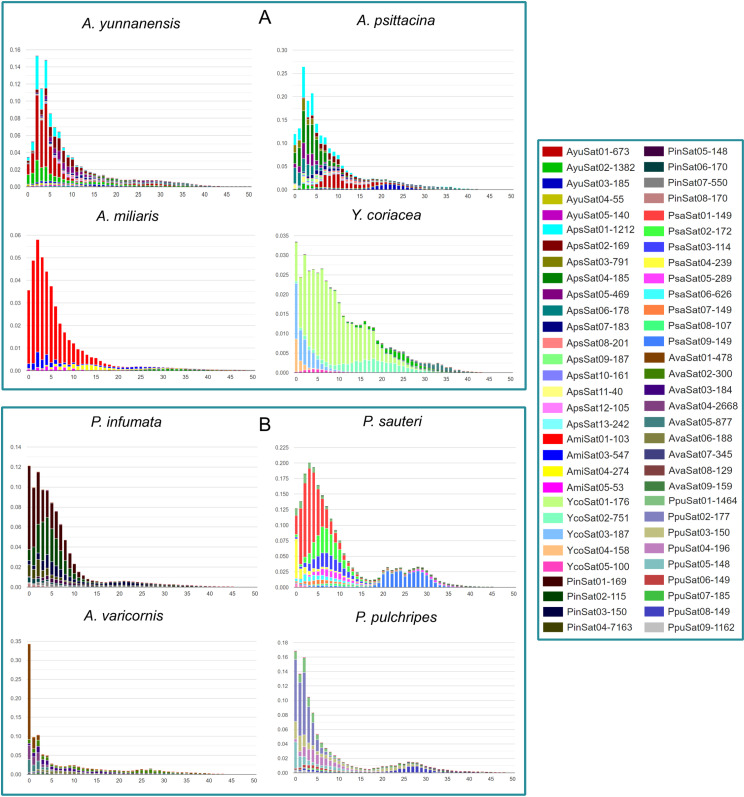
Satellitome divergence landscape in Pyrgomorphidae and Acrididae. (A) The satellite DNA divergence landscapes of family Pyrgomorphidae with y-axis showing the genome proportion occupied by different satDNA sequences and x-axis indicates the Kimura divergence%, which measures the divergence of satDNA from their respective consensus sequences based on the Kimura distance. Lower Kimura substitution levels indicate less divergence and therefore more recent copies, while higher levels suggest older, more diverged sequences. (B) The satellite DNA divergence landscapes of family Acrididae.

The satellitome landscapes revealed significant differences in the composition and evolutionary history of satDNA. In general, the satDNA sequences formed clusters on the left side of the graph at lower Kimura substitution levels, giving an L-shaped satellitome landscape. This indicates a little deviation from their consensus sequence, which confirms that satDNA repeats are recently inserted copies of nucleotides in the genomic landscapes of these species (Fig 6). The species like *A. miliaris*, *A. yunnanensis*, *P. infumata*, and *A. varicornis* predominantly feature satDNA has shown divergence peaks from 0–5% K2P levels, indicating that families of repeats found are younger and the ongoing homogenization process. In contrast, the sequences in *P. sauteri*, *Y. coriacea*, and *P. pulchripes* are distributed from the left to the right side of the graph, with higher Kimura substitution levels of 15–35%. This distribution represents both younger and older, more degenerated copies (Fig 6).

The satellite repeats in *A. miliaris* shows a dominant contribution from a single satDNA family (AmiSat01–103), indicating a less diverse satDNA composition, while species like *P. sauteri*, *A. psittacina*, and *P. pulchripes* exhibit more complex repeat landscapes with multiple peaks, indicating the presence of various repeat families of different ages. Satellite DNA families such as PsaSat08–107 and PsaSat09–149 in *P. sauteri*, YcoSat02–751 in *Y. coriacea*, and PpuSat08–149 in *P. pulchripes* indicate a broader range of ages, with some recent and some older copies in their respective landscapes (Fig 6). The satellitome landscapes across the eight species provide important insights into their genome composition and evolutionary history, highlighting the importance of satDNA in genomic diversity and adaptability.

### Phylogenetic analysis

Phylogenetic analysis based on 13 mitochondrial protein-coding genes (PCGs) and 2 rRNA sequences revealed distinct phylogenetic relationships among the species of the Pyrgomorphidae and Acrididae families. The phylogenetic tree showed a clear division into two clades, one for each family, emphasizing the significant genetic distance between them (Fig 1A). Within the Pyrgomorphidae family, *A. yunnanensis* and *A. psittacina*, belonging to the same genus, were grouped together, reflecting their close evolutionary relationship. Similarly, the Acrididae family formed two sub-groups, clustering related species together. Notably, species grouped together in the phylogenetic tree also exhibited similar repeat landscapes in the comparative repeatome visualization (Figs 1A and 3). This finding suggests that closely related species share relative evolutionary pathways, likely shaped by parallel genomic mechanisms and common evolutionary pressures. These results highlight the critical role of repetitive elements in driving the evolutionary trajectories and genomic architecture of these grasshopper species.

**Fig 7:**
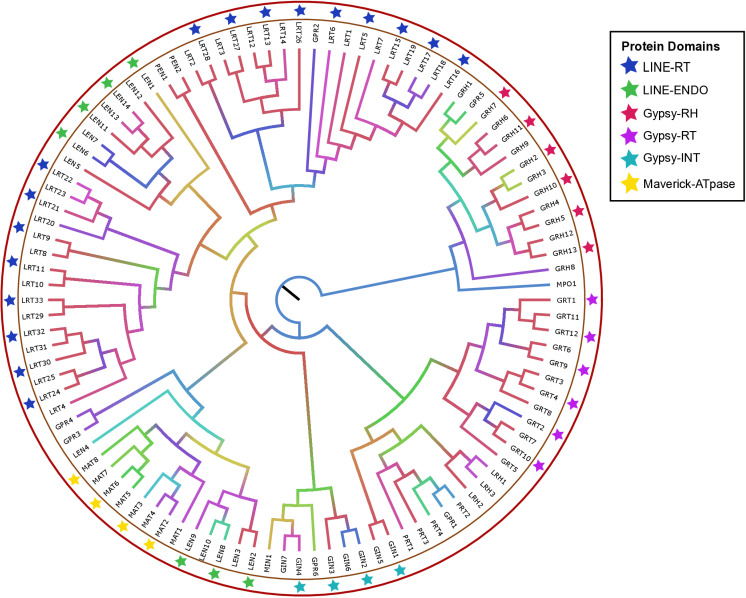
Phylogenetic tree of protein-domain based transposable element (TE) sequences. The maximum likelihood phylogenetic tree illustrates the distinct clustering of various TEs based on their protein-domain consensus sequences, represented by the distinctive star colors. The phylogenetic tree uses a gradient color scheme to represent bootstrap values, where red indicates the highest support (100), transitioning through purple, blue, green, yellow, and orange (31) as bootstrap values decrease.

We identified various protein domains, including RNase-H (RH), reverse transcriptase (RT), endonuclease (ENDO), protease (PROT), integrase (INT), ATPase (AT), and polymerase (POL), in the transposable element consensus sequences of eight grasshopper species. These domains, associated with repetitive DNA elements such as LINE-RT (LRT), LINE-RH (LRH), Gypsy-RT (GRT), and Penelope-ENDO (PEN), play critical roles in the transposition process. To investigate the evolutionary relationships among repetitive elements and their protein domain sequences, we constructed a phylogenetic tree. The tree revealed a clear pattern of domain-specific clustering, reflecting distinct evolutionary pathways. LINE elements (LRT, LRH, LEN) formed tightly grouped clusters, indicating strong evolutionary relationships within this TE type. LINE elements with RH and ENDO domains further formed distinct, well-supported clusters (Fig 7). LTR/Gypsy elements (GRT, GRH, GIN) were also divided into several subclades, while Penelope (PRT, PEN) and Maverick (MAT, MIN) elements grouped into separate, well-supported clades based on their protein domains, demonstrating significant evolutionary conservation (Fig 7).

These findings emphasize the distinct clustering patterns among domain types within repeats such as LINE, Penelope, LTR/Gypsy, and Maverick elements, highlighting their independent evolutionary pathways. This study offers valuable insights into the evolution of TEs in grasshoppers and their role in contributing to genomic diversity.

## Discussion

### Relationship between repetitive DNA and genome size variation

Orthoptera stands out as the only group of insects with expanded genome sizes (GS), exhibiting a remarkable 20-fold variation. The smallest genome size (0.95 pg) measured within this group is in *Oecanthus euryelytra* Ichikawa (Ensifera: Gryllidae), while the largest (21.96 pg) is in *Bryodemella tuberculata* Fabricius (Caelifera: Acrididae) [21,22]. Recent studies have suggested that an increase in repetitive elements is significantly associated with larger genome sizes, contributing substantially to genomic evolution. In the Animal groups that lack genome duplication events, repetitive sequences are the major contributors in genome size variation [48,49]. In the current study, we identified and quantified the repetitive content in eight Orthoptera species and conducted a comparative repeatome analysis to understand the evolutionary dynamics of repetitive elements into genome size variation. Our findings showed a higher repetitive content, ranging from 51% to 74% of the genome, confirming previous investigations that Orthoptera genomes, such as those of *S. gregaria*, *L. migratoria*, *V. viatica*, species from the genus *Calliptamus*, and Gomphocerine grasshoppers, are rich in repetitive sequences [26,29,34,50]. This suggests that repetitive elements could play a crucial role in the genome size variation.

Given the aim to investigate the influence of repetitive DNA on genome size variation, we measured genome sizes using flow cytometry, which ranged from 7.67 to 18.612 pg (Fig 1B and Table 1). The RE2 results corroborated this relationship, showing that species with smaller genome sizes, such as 7.67 pg, 8.382 pg, and 9.69 pg in *A. psittacina*, *A. yunnanensis*, and *P. pulchripes*, respectively, exhibited lower repetitive content at 58.27%, 67.12%, and 67.17% (Fig 1D and Table 1). These findings support previous reports that repetitive elements, particularly TEs, contribute significantly to genome size expansion in insects [20]. In particular, *A. miliaris*, which had the largest genome (18.612 pg) in our study, showed a substantial contribution from transposable elements, with LINEs alone accounting for 54% of the total repetitive content. However, the overall repeat content was 50.75%, suggesting that genome expansion in *A. miliaris* may have been driven by the amplification of specific TE families rather than a uniform increase across all repeat types. To strengthen confidence in the repeat annotations generated by RepeatExplorer2, we performed additional validation steps. BLAST searches against the NCBI nt database showed no significant similarity between the identified TAREAN consensus sequences and human, plant, or bacterial DNA, effectively ruling out contamination. Additionally, mapping these sequences to raw NGS reads using RepeatProfiler produced consistent, well-covered repeat profiles across species (S5 and S6 Figs in S1 File), confirming their genomic presence. These results align with previous studies that used RepeatProfiler for similar purposes [13,20], reinforcing the reliability of RepeatExplorer2 outputs in species lacking high-quality reference genomes.

The correlation analysis between repetitive DNA content (Pearson correlation: r = 0.943, p = 4.449e-04) and genome size, as well as TE content (r = 0.923, p = 1.069e-03) and genome size, revealed a strong positive correlation (Fig 2). Similarly, the correlation analysis between genome size and major repeat types, including satDNA, showed moderate to strong positive correlations, except for rDNA, which is present in very low proportions within the genome (S2 Fig in S1 File). A similar trend of repeat content association with genome size has been reported in previous studies on insects, particularly within grasshopper species [28,36,37,51]. These findings underscore the significance of repetitive DNA in genome size variation, highlighting the complex evolutionary dynamics shaping Orthoptera genomes.

### Repeatome composition in pyrgomorphidae and acrididae

The overall composition of repeat elements across the studied species revealed a conserved repeatome structure, suggesting shared evolutionary histories. Major repeat types identified include non-LTR retrotransposons such as LINE and Penelope, LTR-retrotransposon lineages including Ty3_gypsy, Ty1-copia, Retrovirus and Bel-Pao, along with DNA transposons like Helitron and Maverick (Fig 1D). As previously reported, the relatively short timescale of divergence among these species may account for the overall similarity in their repeatome composition [28,29]. Furthermore, the comparative analysis showed that many of the most abundant repeat clusters are shared among closely related species within the same genus, such as *A. yunnanensis* and *A. psittacina* (Fig 3), and also across families. Despite this overall similarity, a small proportion of species-specific repeats was also identified, particularly satellite DNAs, which are considered among the most dynamic and rapidly evolving components of eukaryotic genomes [52–54].

Although LINE elements are the most abundant repeats overall, the family Pyrgomorphidae showed a relatively higher proportion of these elements (28–54%) compared to Acrididae (24–25%) of the total repetitive content (Fig 1D). We observed that LINE and Ty3_gypsy elements are generally more abundant and form relatively larger clusters across species, suggesting their active proliferation, diversification, and amplification in Orthoptera genomes [28,34,37]. This amplification may contribute to genome size variation and influence other evolutionary mechanisms in these species.

### Evolutionary dynamics of TE divergence activity

Considering that genome size in Pyrgomorphidae and Acrididae is significantly influenced by TE expansion, we analyzed TE insertion activity in the studied genomes to determine whether these invasions represent recent burst events or ongoing ancient TE proliferation processes. The Kimura-based divergence landscapes indicated that TE accumulations are primarily from recent to near-past insertions, which have substantially contributed to genome expansion in Orthoptera genomes [20,36,37]. The repeat landscapes in Pyrgomorphidae exhibited a bell-curve pattern, reflecting relatively lower TE activity in the most recent times, along with evidence of both recent past and ancient activity. However, *Y. coriacea* showed distinct peaks at lower Kimura levels, indicating younger TE insertions (Fig 4). In contrast, the genomic landscapes in Acrididae revealed substantial TE expansion, characterized by more recent bursts of major TEs, including LINEs, LTRs, and DNA transposons. The variation in TE accumulation observed among these genomes can likely be attributed to TE-driven evolutionary pressures throughout the evolutionary history of grasshopper species [30,48]. Our study confirms that both recent and ancient TE divergence patterns drive genome expansion and complexity in Pyrgomorphidae and Acrididae.

### Comparative satellitome analysis reveals different satDNA families

Satellite repeats constitute a considerable part of the repetitive DNA sequences in eukaryotic genomes. It has been observed that a multiple range of satDNA families are identified in various insect species but often contain a fewer genome proportion [55,56]. For instance, 60 satDNA families were reported in *Locusta migratoria*, 58 in *Oedaleus decorus* [35], 45 families in *Eneoptera surinamensis* [56], 16 in *Drosophila melanogaster* [13] and 76 satellite DNA families were identified in *Pyrgomorpha conica* [57]. Previous comparative satellitome studies have also identified 188 satDNA families in 37 *D. melanogaster* species [58], 129 families in *Vandiemenella viatica* morabine grasshopper’s four races [34], and 20 families in 3 *Calliptamus* species [29]. Similarly, in the current study, we identified 65 satDNA families from eight species of grasshoppers using the RE2 comparative clustering platform. Most of these satDNA families were shared among all the species. We also discovered some species-specific satDNA families that may contribute to genome size expansion, in agreement with previously reported findings, although a few studies suggest that satDNA might not have any role in genome size expansion [28,29].

### Satellite DNA abundance, monomer length variation and divergence on satellitome landscapes

Satellite DNA content can vary widely even among closely related species, reflecting ongoing processes of amplification and loss over evolutionary time [59]. In our study, satDNA abundance differed notably across genomes, consistent with previous observations of lower satellite DNA content in insects [53,57]. Among the 65 identified families, PsaSat01 was notably dominant in the *P. sauteri* genome (4.39%), while only five families were consistently shared across all eight species, suggesting the lineage-specific nature of satellite DNA evolution (Fig 5). This pattern may also indicate that evolutionary pressures do not favor the long-term retention of most satellite sequences, allowing only a few to remain conserved in closely related genomes [60]. As reported in earlier studies [29,58], satDNA consensus sequences were predominantly AT-rich, with A + T content in our study ranging from 47.3% to 76.19% (median: 59.27%). The exception was the PpuSat05 family, which had the highest G + C content at 52.7%.

The monomer length of satDNA families varied widely among Orthoptera genomes. In this study, monomer lengths ranged from 21 nt in the AmiSat02 family (*A. miliaris*) to 7163 nt in the PinSat04 family (*P. infumata*). Notably, PinSat04 (7.163 kb) and AvaSat04 (2.668 kb) were the largest satDNA families identified, surpassing the previously reported CSat13–2150 (2.150 kb) [29]. Similarly, large satDNA monomer lengths have been documented in other insect species, including 2.5 kb in the ant *Monomorium subopacum* [61], 2 kb (HvarSat07–2000) in *Hippodamia variegata* [62], 956 nt (RferSat26–956) in *Rhynchophorus ferrugineus* [63], and 980 nt (RproSat22–980) in *Rhodnius prolixus* [64].

The divergence patterns observed in satellitome landscapes provide insight into the evolutionary dynamics satellite DNA families. The satellitome landscapes showed a lower trend of Kimura divergence levels in species-specific satDNA families. For example, AyuSat01 indicated a divergence of only 4.80% in *A. yunnanensis*, while same satDNA family (AyuSat01) showed significantly higher divergence rate of 12.27% in *A. psittacine* (Fig 6 and S4 Fig in S1 File). This suggests that satellite repeats are evolutionarily dynamic part of the eukaryotic genome, which perform a significant role in genomic evolution, regulation, and speciation [65,66]. The K2P divergence of satDNA is inversely related to amplification and homogenization processes, while directly linked to mutation rates in the genome [62]. Lower divergence rates in satDNA families indicate ongoing homogenization, as seen in the clustering of most sequences on the left side of the graph with divergence levels below 5%, representing newer satellite copies. Notably, satDNA families such as PsaSat08–107 and PsaSat09–149 in *P. sauteri* and YcoSat02–751 in *Y. coriacea* displayed a broader age distribution and divergence, confirming the presence of both younger and older copies in their respective satellitome landscapes (Fig 6). These findings of satDNA peaks at lower Kimura levels align with previous studies in insects, particularly within the Orthoptera group [28,29,34,57,67].

### Phylogenetic analysis

The maximum likelihood phylogenetic tree, constructed using 15 mitochondrial genes extracted through MitoFinder, revealed a clear division into two major clades, each representing species from one family (Fig 1A). This distinct split underscore the distant phylogenetic relationship between the two families. Interestingly, closely related species on the phylogenetic tree, such as *A. yunnanensis* and *A. psittacina*, also exhibited similar repeat landscapes (Fig 1A and Fig 3). This observation highlights the evolutionary role of TEs in shaping genomic architecture [68] and underscores the relationship between phylogenetics and repetitive DNA in driving the evolution and genomic complexity of grasshopper species.

Various protein domains, including RNase-H (RH), reverse transcriptase (RT), endonuclease (ENDO), integrase (INT), protease (PROT), ATPase (AT), and polymerase (POL), were identified in the TE consensus sequences. These domains are associated with specific repetitive elements, such as LINE-RT (LRT), Gypsy-RH (GRH), and Penelope-ENDO (PEN), which play crucial roles in the integration and replication of retroelements within the genome [69]. While previous phylogenetic studies have focused on RT, RH, and INT domains associated with LTR-retrotransposons in plant species [70,71], no similar analyses have been conducted for TE protein domain-based sequences in Orthoptera insects. In this study, we included all identified domains, associated with LTR-retrotransposons (Ty3_gypsy), non-LTR elements (LINE and Penelope), and DNA transposons (Maverick) to explore their phylogenetic relationships. The phylogenetic tree revealed distinct evolutionary lineages for different TE types, with each repeat type forming separate clades based on their specific domains (Fig 7), indicating unique evolutionary trajectories for TEs.

The multiple evolutionary pathways revealed in the phylogenetic tree indicate domain-specific transposition mechanisms in TEs, which can be used for the further classification of LTR-retrotransposons (Ty3_gypsy, Ty1_copia) into their respective subfamilies, driven by their higher sequence diversity [70,72]. This study provides insights into the development of a phylogenetic analysis-based classification system for major TE families in Orthoptera genomes, offering a deeper understanding of their evolutionary dynamics.

## Conclusions

The genome sizes of the eight grasshopper species are found to be exceptionally large, ranging from 7.670 pg to 18.612 pg, along with a higher proportion of repetitive DNA elements. The repetitive DNA is ranged from 51% to 74%, with a mean value of 64.26% of the total genomic content. Repeats such as LINE, satDNA, Ty3_Gypsy, and Penelope were found in higher proportions, likely contributing significantly to genome gigantism. For instance, in our study, the largest genome, 18.612 pg in *A. miliaris*, contained LINE elements constituting more than half (54%) of its total repetitive content. The correlation analysis between genome size and repeat elements, including TEs and satDNA, revealed a strong positive relationship, suggesting that repeat elements, particularly TEs, may have contributed to the expanded genome sizes observed in Pyrgomorphidae and Acrididae. Comparative repetitive analysis suggested that TEs are mostly conserved repeats with shared regions, while satDNA consisted mostly of species-specific regions, reaffirming the evolutionary dynamic nature of satellite repeats. Additionally, satellitome analysis revealed 65 satDNA families across the eight species, which are mostly mapped to lower Kimura divergence levels (<5%), indicating that newer copies are present in the genome. We also performed phylogenetic analysis on protein domain-based TE consensus sequences, demonstrating that sequences from the same protein domain and TEs tend to cluster together, suggesting domain-specific evolutionary pathways in the genome. Our study provides a strong foundation for further genomic and evolutionary research on the repetitive-rich, gigantic genomes of Orthoptera. Long-read sequencing technologies such as Oxford Nanopore, PacBio, and Hi-C data are required to produce a high-quality chromosome-level assemblies and further repeatome investigations in these fascinating grasshopper species.

## Supporting information

S1 File S1 Fig. Clustering summary of repetitive elements across eight species. A graphical summary generated by RE2, with the left panel showing repetitive reads and the right-side box displaying reads analyzed as singlets. S2 Fig. Correlation analysis between genome size and major repetitive elements across species. Distinctive colored lines represent the correlation relationships between genome size and major repeat elements, including LINEs, LTRs, Penelope, DNA transposons, rDNA, satellites, and unclassified repeats. S3 Fig. Structure of different satellite repeat clusters. Unique structures of satDNA clusters and their proportions across various species. S4 Fig. Satellite DNA abundance percentages in eight species of Pyrgomorphidae and Acrididae. The abundance percentages of satellite repeats in selected species of Pyrgomorphidae and Acrididae, calculated using RepeatMasker. S5 Fig. Color-enhanced repeat profiles of top clusters in Pyrgomorphidae species. The x-axis represents the position along each consensus sequence, while the y-axis indicates the coverage depth at each position. S6 Fig. Color-enhanced repeat profiles of top clusters in Acrididae species. The x-axis represents the position along each consensus sequence, while the y-axis indicates the coverage depth at each position.
(PDF)

S2 File S1 Table. Specimen details for the species used in the experiment. S2 Table. Number of reads sampled for repetitive sequence analysis in each species. S3 Table. The prefixes used for the selected eight species. S4 Table. Types and proportions (%) of repetitive DNA identified in the eight species. S5 Table. RepeatMasker results for AmiSat02 using monomer to pentamer units in A. miliaris.
(XLSX)

## References

[1] Charlesworth B, Sniegowski P, Stephan W. The evolutionary dynamics of repetitive DNA in eukaryotes. Nature. 1994;371(6494):215–20. doi: 10.1038/371215a0 8078581

[2] Kim YB, Oh JH, McIver LJ, Rashkovetsky E, Michalak K, Garner HR, et al. Divergence of Drosophila melanogaster repeatomes in response to a sharp microclimate contrast in Evolution Canyon, Israel. Proc Natl Acad Sci U S A. 2014;111(29):10630–5. doi: 10.1073/pnas.1410372111 25006263 PMC4115526

[3] Thakur J, Packiaraj J, Henikoff S. Sequence, Chromatin and Evolution of Satellite DNA. Int J Mol Sci. 2021;22(9):4309. doi: 10.3390/ijms22094309 33919233 PMC8122249

[4] Louzada S, Lopes M, Ferreira D, Adega F, Escudeiro A, Gama-Carvalho M, et al. Decoding the Role of Satellite DNA in Genome Architecture and Plasticity-An Evolutionary and Clinical Affair. Genes (Basel). 2020;11(1):72. doi: 10.3390/genes11010072 31936645 PMC7017282

[5] Richard G-F, Kerrest A, Dujon B. Comparative genomics and molecular dynamics of DNA repeats in eukaryotes. Microbiol Mol Biol Rev. 2008;72(4):686–727. doi: 10.1128/MMBR.00011-08 19052325 PMC2593564

[6] Khost DE, Eickbush DG, Larracuente AM. Single-molecule sequencing resolves the detailed structure of complex satellite DNA loci in Drosophila melanogaster. Genome Res. 2017;27(5):709–21. doi: 10.1101/gr.213512.116 28373483 PMC5411766

[7] Dernburg AF, Sedat JW, Hawley RS. Direct evidence of a role for heterochromatin in meiotic chromosome segregation. Cell. 1996;86(1):135–46. doi: 10.1016/s0092-8674(00)80084-7 8689681

[8] Kuhn GCS. “Satellite DNA transcripts have diverse biological roles in Drosophila”. Heredity (Edinb). 2015;115(1):1–2. doi: 10.1038/hdy.2015.12 25806543 PMC4815497

[9] Shatskikh AS, Kotov AA, Adashev VE, Bazylev SS, Olenina LV. Functional Significance of Satellite DNAs: Insights From Drosophila. Front Cell Dev Biol. 2020;8:312. doi: 10.3389/fcell.2020.00312 32432114 PMC7214746

[10] Wallrath LL, Rodriguez-Tirado F, Geyer PK. Shining Light on the Dark Side of the Genome. Cells. 2022;11(3):330. doi: 10.3390/cells11030330 35159140 PMC8834555

[11] Ferreira D, Meles S, Escudeiro A, Mendes-da-Silva A, Adega F, Chaves R. Satellite non-coding RNAs: the emerging players in cells, cellular pathways and cancer. Chromosome Res. 2015;23(3):479–93. doi: 10.1007/s10577-015-9482-8 26293605

[12] Ugarković Đ, Sermek A, Ljubić S, Feliciello I. Satellite DNAs in Health and Disease. Genes (Basel). 2022;13(7):1154. doi: 10.3390/genes13071154 35885937 PMC9324158

[13] Ruiz-Ruano FJ, López-León MD, Cabrero J, Camacho JPM. High-throughput analysis of the satellitome illuminates satellite DNA evolution. Sci Rep. 2016;6:28333. doi: 10.1038/srep28333 27385065 PMC4935994

[14] Bourque G, Burns KH, Gehring M, Gorbunova V, Seluanov A, Hammell M, et al. Ten things you should know about transposable elements. Genome Biol. 2018;19(1):199. doi: 10.1186/s13059-018-1577-z 30454069 PMC6240941

[15] Wicker T, Sabot F, Hua-Van A, Bennetzen JL, Capy P, Chalhoub B, et al. A unified classification system for eukaryotic transposable elements. Nat Rev Genet. 2007;8(12):973–82. doi: 10.1038/nrg2165 17984973

[16] Barro-Trastoy D, Köhler C. Helitrons: genomic parasites that generate developmental novelties. Trends Genet. 2024;40(5):437–48. doi: 10.1016/j.tig.2024.02.002 38429198

[17] Wells JN, Feschotte C. A Field Guide to Eukaryotic Transposable Elements. Annu Rev Genet. 2020;54:539–61. doi: 10.1146/annurev-genet-040620-022145 32955944 PMC8293684

[18] Gregory TR. Coincidence, coevolution, or causation? DNA content, cellsize, and the C‐value enigma. Biological Reviews. 2001;76(1):65–101. doi: 10.1111/j.1469-185x.2000.tb00059.x 11325054

[19] Jing X, Liu X, Yuan H, Dai Y, Zheng Y, Zhao L, et al. Evolutionary dynamics of genome size and transposable elements in crickets (Ensifera: Gryllidea). Systematic Entomology. 2024;49(4):549–64. doi: 10.1111/syen.12629

[20] Liu X, Majid M, Yuan H, Chang H, Zhao L, Nie Y, et al. Transposable element expansion and low-level piRNA silencing in grasshoppers may cause genome gigantism. BMC Biol. 2022;20(1):243. doi: 10.1186/s12915-022-01441-w 36307800 PMC9615261

[21] Yuan H, Huang Y, Mao Y, Zhang N, Nie Y, Zhang X, et al. The Evolutionary Patterns of Genome Size in Ensifera (Insecta: Orthoptera). Front Genet. 2021;12:693541. doi: 10.3389/fgene.2021.693541 34249107 PMC8261143

[22] Hawlitschek O, Sadílek D, Dey L-S, Buchholz K, Noori S, Baez IL, et al. New estimates of genome size in Orthoptera and their evolutionary implications. PLoS One. 2023;18(3):e0275551. doi: 10.1371/journal.pone.0275551 36920952 PMC10016648

[23] Sun C, López Arriaza JR, Mueller RL. Slow DNA loss in the gigantic genomes of salamanders. Genome Biol Evol. 2012;4(12):1340–8. doi: 10.1093/gbe/evs103 23175715 PMC3542557

[24] Bensasson D, Petrov DA, Zhang DX, Hartl DL, Hewitt GM. Genomic gigantism: DNA loss is slow in mountain grasshoppers. Mol Biol Evol. 2001;18(2):246–53. doi: 10.1093/oxfordjournals.molbev.a003798 11158383

[25] Kapusta A, Suh A, Feschotte C. Dynamics of genome size evolution in birds and mammals. Proc Natl Acad Sci U S A. 2017;114(8):E1460–9. doi: 10.1073/pnas.1616702114 28179571 PMC5338432

[26] Naville M, Henriet S, Warren I, Sumic S, Reeve M, Volff J-N, et al. Massive Changes of Genome Size Driven by Expansions of Non-autonomous Transposable Elements. Curr Biol. 2019;29(7):1161-1168.e6. doi: 10.1016/j.cub.2019.01.080 30880010

[27] Ustinova J, Achmann R, Cremer S, Mayer F. Long repeats in a huge genome: microsatellite loci in the grasshopper Chorthippus biguttulus. J Mol Evol. 2006;62(2):158–67. doi: 10.1007/s00239-005-0022-6 16474983

[28] Shah A, Hoffman JI, Schielzeth H. Comparative Analysis of Genomic Repeat Content in Gomphocerine Grasshoppers Reveals Expansion of Satellite DNA and Helitrons in Species with Unusually Large Genomes. Genome Biol Evol. 2020;12(7):1180–93. doi: 10.1093/gbe/evaa119 32539114 PMC7486953

[29] Majid M, Yuan H. Comparative Analysis of Transposable Elements in Genus Calliptamus Grasshoppers Revealed That Satellite DNA Contributes to Genome Size Variation. Insects. 2021;12(9):837. doi: 10.3390/insects12090837 34564277 PMC8466570

[30] Margulies M, Egholm M, Altman WE, Attiya S, Bader JS, Bemben LA, et al. Genome sequencing in microfabricated high-density picolitre reactors. Nature. 2005;437(7057):376–80. doi: 10.1038/nature03959 16056220 PMC1464427

[31] Peona V, Weissensteiner MH, Suh A. How complete are “complete” genome assemblies?-An avian perspective. Mol Ecol Resour. 2018;18(6):1188–95. doi: 10.1111/1755-0998.12933 30035372

[32] Weiss-Schneeweiss H, Leitch A, McCann J, Jang T-S, Macas J. Employing next generation sequencing to explore the repeat landscape of the plant genome. Next Gener Seq Plant Syst Regnum Veg. 2015;157:155–9.

[33] Macas J, Neumann P, Navrátilová A. Repetitive DNA in the pea (Pisum sativum L.) genome: comprehensive characterization using 454 sequencing and comparison to soybean and Medicago truncatula. BMC Genomics. 2007;8:427. doi: 10.1186/1471-2164-8-427 18031571 PMC2206039

[34] Palacios-Gimenez OM, Koelman J, Palmada-Flores M, Bradford TM, Jones KK, Cooper SJB, et al. Comparative analysis of morabine grasshopper genomes reveals highly abundant transposable elements and rapidly proliferating satellite DNA repeats. BMC Biol. 2020;18(1):199. doi: 10.1186/s12915-020-00925-x 33349252 PMC7754599

[35] Camacho JPM, Cabrero J, López-León MD, Martín-Peciña M, Perfectti F, Garrido-Ramos MA, et al. Author Correction: Satellitome comparison of two oedipodine grasshoppers highlights the contingent nature of satellite DNA evolution. BMC Biol. 2022;20(1):69. doi: 10.1186/s12915-022-01260-z 35317788 PMC8941793

[36] Zhao L, Yuan H, Liu X, Chang H, Jing X, Nie Y, et al. Evolutionary dynamics of repetitive elements and their relationship with genome size in Acrididae. Genomics. 2025;117(1):110971. doi: 10.1016/j.ygeno.2024.110971 39643065

[37] Nie Y, Liu X, Zhao L, Huang Y. Repetitive element expansions contribute to genome size gigantism in Pamphagidae: A comparative study (Orthoptera, Acridoidea). Genomics. 2024;116(5):110896. doi: 10.1016/j.ygeno.2024.110896 39025318

[38] Song H, Mariño-Pérez R, Woller DA, Cigliano MM. Evolution, Diversification, and Biogeography of Grasshoppers (Orthoptera: Acrididae). Insect Systematics and Diversity. 2018;2(4). doi: 10.1093/isd/ixy008

[39] Akwanjoh SR, Tita NM. First Ever Data on the Occurrences of Acrididae (Acridoidea: Orthoptera) Grasshoppers in the North West Region of Cameroon. JBLS. 2020;11(1):154. doi: 10.5296/jbls.v11i1.16432

[40] MARIÑO‐PÉREZ R, SONG H. Phylogeny of the grasshopper family Pyrgomorphidae (Caelifera, Orthoptera) based on morphology. Systematic Entomology. 2017;43(1):90–108. doi: 10.1111/syen.12251

[41] Lower SS, Johnston JS, Stanger-Hall KF, Hjelmen CE, Hanrahan SJ, Korunes K, et al. Genome Size in North American Fireflies: Substantial Variation Likely Driven by Neutral Processes. Genome Biol Evol. 2017;9(6):1499–512. doi: 10.1093/gbe/evx097 28541478 PMC5499882

[42] Dolezel J, Bartos J, Voglmayr H, Greilhuber J. Nuclear DNA content and genome size of trout and human. Cytometry A. 2003;51(2):127–8; author reply 129. doi: 10.1002/cyto.a.10013 12541287

[43] Dolezel J, Greilhuber J. Nuclear genome size: are we getting closer?. Cytometry A. 2010;77(7):635–42. doi: 10.1002/cyto.a.20915 20583277

[44] Novák P, Neumann P, Macas J. Graph-based clustering and characterization of repetitive sequences in next-generation sequencing data. BMC Bioinformatics. 2010;11:378. doi: 10.1186/1471-2105-11-378 20633259 PMC2912890

[45] Treangen TJ, Salzberg SL. Repetitive DNA and next-generation sequencing: computational challenges and solutions. Nat Rev Genet. 2011;13(1):36–46. doi: 10.1038/nrg3117 22124482 PMC3324860

[46] Novák P, Neumann P, Pech J, Steinhaisl J, Macas J. RepeatExplorer: a Galaxy-based web server for genome-wide characterization of eukaryotic repetitive elements from next-generation sequence reads. Bioinformatics. 2013;29(6):792–3. doi: 10.1093/bioinformatics/btt054 23376349

[47] Liu X, Zhao L, Majid M, Huang Y. Orthoptera-TElib: a library of Orthoptera transposable elements for TE annotation. Mob DNA. 2024;15(1):5. doi: 10.1186/s13100-024-00316-x 38486291 PMC10941475

[48] Gregory TR. Synergy between sequence and size in large-scale genomics. Nat Rev Genet. 2005;6(9):699–708. doi: 10.1038/nrg1674 16151375

[49] Petrov DA. Evolution of genome size: new approaches to an old problem. Trends Genet. 2001;17(1):23–8. doi: 10.1016/s0168-9525(00)02157-0 11163918

[50] Wang X, Fang X, Yang P, Jiang X, Jiang F, Zhao D, et al. The locust genome provides insight into swarm formation and long-distance flight. Nat Commun. 2014;5:2957. doi: 10.1038/ncomms3957 24423660 PMC3896762

[51] Xu Y, Tang Y, Feng W, Yang Y, Cui Z. Comparative Analysis of Transposable Elements Reveals the Diversity of Transposable Elements in Decapoda and Their Effects on Genomic Evolution. Mar Biotechnol (NY). 2023;25(6):1136–46. doi: 10.1007/s10126-023-10265-w 37923816

[52] Mehrotra S, Goyal V. Repetitive sequences in plant nuclear DNA: types, distribution, evolution and function. Genomics Proteomics Bioinformatics. 2014;12(4):164–71. doi: 10.1016/j.gpb.2014.07.003 25132181 PMC4411372

[53] Haq IU, Muhammad M, Yuan H, Ali S, Abbasi A, Asad M, et al. Satellitome Analysis and Transposable Elements Comparison in Geographically Distant Populations of Spodoptera frugiperda. Life (Basel). 2022;12(4):521. doi: 10.3390/life12040521 35455012 PMC9026859

[54] Ferretti ABSM, Milani D, Palacios-Gimenez OM, Ruiz-Ruano FJ, Cabral-de-Mello DC. High dynamism for neo-sex chromosomes: satellite DNAs reveal complex evolution in a grasshopper. Heredity (Edinb). 2020;125(3):124–37. doi: 10.1038/s41437-020-0327-7 32499661 PMC7426270

[55] Palomeque T, Lorite P. Satellite DNA in insects: a review. Heredity (Edinb). 2008;100(6):564–73. doi: 10.1038/hdy.2008.24 18414505

[56] Palacios-Gimenez OM, Dias GB, de Lima LG, Kuhn GCES, Ramos É, Martins C, et al. High-throughput analysis of the satellitome revealed enormous diversity of satellite DNAs in the neo-Y chromosome of the cricket Eneoptera surinamensis. Sci Rep. 2017;7(1):6422. doi: 10.1038/s41598-017-06822-8 28743997 PMC5527012

[57] Ruiz-Ruano FJ, Castillo-Martínez J, Cabrero J, Gómez R, Camacho JPM, López-León MD. High-throughput analysis of satellite DNA in the grasshopper Pyrgomorpha conica reveals abundance of homologous and heterologous higher-order repeats. Chromosoma. 2018;127(3):323–40. doi: 10.1007/s00412-018-0666-9 29549528

[58] de Lima LG, Ruiz-Ruano FJ. In-Depth Satellitome Analyses of 37 Drosophila Species Illuminate Repetitive DNA Evolution in the Drosophila Genus. Genome Biol Evol. 2022;14(5):evac064. doi: 10.1093/gbe/evac064 35511582 PMC9113345

[59] Mestrović N, Plohl M, Mravinac B, Ugarković D. Evolution of satellite DNAs from the genus Palorus--experimental evidence for the “library” hypothesis. Mol Biol Evol. 1998;15(8):1062–8. doi: 10.1093/oxfordjournals.molbev.a026005 9718733

[60] Dos Santos RZ, Calegari RM, Silva DMZ de A, Ruiz-Ruano FJ, Melo S, Oliveira C, et al. A Long-Term Conserved Satellite DNA That Remains Unexpanded in Several Genomes of Characiformes Fish Is Actively Transcribed. Genome Biol Evol. 2021;13(2):evab002. doi: 10.1093/gbe/evab002 33502491 PMC8210747

[61] Lorite P, Carrillo JA, Aguilar JA, Palomeque T. Isolation and characterization of two families of satellite DNA with repetitive units of 135 bp and 2.5 kb in the ant Monomorium subopacum (Hymenoptera, Formicidae). Cytogenet Genome Res. 2004;105(1):83–92. doi: 10.1159/000078013 15218262

[62] Mora P, Vela J, Ruiz-Ruano FJ, Ruiz-Mena A, Montiel EE, Palomeque T, et al. Satellitome Analysis in the Ladybird Beetle Hippodamia variegata (Coleoptera, Coccinellidae). Genes (Basel). 2020;11(7):783. doi: 10.3390/genes11070783 32668664 PMC7397073

[63] Montiel EE, Mora P, Rico-Porras JM, Palomeque T, Lorite P. Satellitome of the Red Palm Weevil, Rhynchophorus ferrugineus (Coleoptera: Curculionidae), the Most Diverse Among Insects. Front Ecol Evol. 2022;10. doi: 10.3389/fevo.2022.826808

[64] Montiel EE, Panzera F, Palomeque T, Lorite P, Pita S. Satellitome Analysis of Rhodnius prolixus, One of the Main Chagas Disease Vector Species. Int J Mol Sci. 2021;22(11):6052. doi: 10.3390/ijms22116052 34205189 PMC8199985

[65] Sproul JS, Khost DE, Eickbush DG, Negm S, Wei X, Wong I, et al. Dynamic Evolution of Euchromatic Satellites on the X Chromosome in Drosophila melanogaster and the simulans Clade. Mol Biol Evol. 2020;37(8):2241–56. doi: 10.1093/molbev/msaa078 32191304 PMC7403614

[66] Samoluk SS, Robledo G, Bertioli D, Seijo JG. Evolutionary dynamics of an at-rich satellite DNA and its contribution to karyotype differentiation in wild diploid Arachis species. Mol Genet Genomics. 2017;292(2):283–96. doi: 10.1007/s00438-016-1271-3 27838847

[67] Majid M, Khan H, Liu X, Shaheer M, Huang Y. Evolutionary Dynamics of Satellite DNA Repeats across the Tettigoniidae Family: Insights from Genomic Analysis. Biomolecules. 2024;14(8):915. doi: 10.3390/biom14080915 39199303 PMC11352069

[68] Colonna Romano N, Fanti L. Transposable Elements: Major Players in Shaping Genomic and Evolutionary Patterns. Cells. 2022;11(6):1048. doi: 10.3390/cells11061048 35326499 PMC8947103

[69] Kojima KK. Structural and sequence diversity of eukaryotic transposable elements. Genes Genet Syst. 2020;94(6):233–52. doi: 10.1266/ggs.18-00024 30416149

[70] Neumann P, Novák P, Hoštáková N, Macas J. Systematic survey of plant LTR-retrotransposons elucidates phylogenetic relationships of their polyprotein domains and provides a reference for element classification. Mob DNA. 2019;10:1. doi: 10.1186/s13100-018-0144-1 30622655 PMC6317226

[71] Llorens C, Muñoz-Pomer A, Bernad L, Botella H, Moya A. Network dynamics of eukaryotic LTR retroelements beyond phylogenetic trees. Biol Direct. 2009;4:41. doi: 10.1186/1745-6150-4-41 19883502 PMC2774666

[72] Aroh O, Halanych KM. Genome-wide characterization of LTR retrotransposons in the non-model deep-sea annelid Lamellibrachia luymesi. BMC Genomics. 2021;22(1):466. doi: 10.1186/s12864-021-07749-1 34157969 PMC8220671

