# Supplementary material for: Comparative repeatome analysis of Pyrgomorphidae and Acrididae (Orthoptera: Caelifera) revealed the contribution of repetitive DNA in genome gigantism
Source: PLoS One. 2025 Jun 2;20(6):e0325165. doi: 10.1371/journal.pone.0325165 (PMC12129174; doi:10.1371/journal.pone.0325165)
Supplement: S1 File — S1 Fig. Clustering summary of repetitive elements across eight species. A graphical summary generated by RE2, with the left panel showing repetitive reads and the right-side box displaying reads analyzed as singlets. S2 Fig. Correlation analysis between genome size and major repetitive elements across species. Distinctive colored lines represent the correlation relationships between genome size and major repeat elements, including LINEs, LTRs, Penelope, DNA transposons, rDNA, satellites, and unclassified repeats. S3 Fig. Structure of different satellite repeat clusters. Unique structures of satDNA clusters and their proportions across various species. S4 Fig. Satellite DNA abundance percentages in eight species of Pyrgomorphidae and Acrididae. The abundance percentages of satellite repeats in selected species of Pyrgomorphidae and Acrididae, calculated using RepeatMasker. S5 Fig. Color-enhanced repeat profiles of top clusters in Pyrgomorphidae species. The x-axis represents the position along each consensus sequence, while the y-axis indicates the coverage depth at each position. S6 Fig. Color-enhanced repeat profiles of top clusters in Acrididae species. The x-axis represents the position along each consensus sequence, while the y-axis indicates the coverage depth at each position. (PDF) [file pone.0325165.s001.pdf]

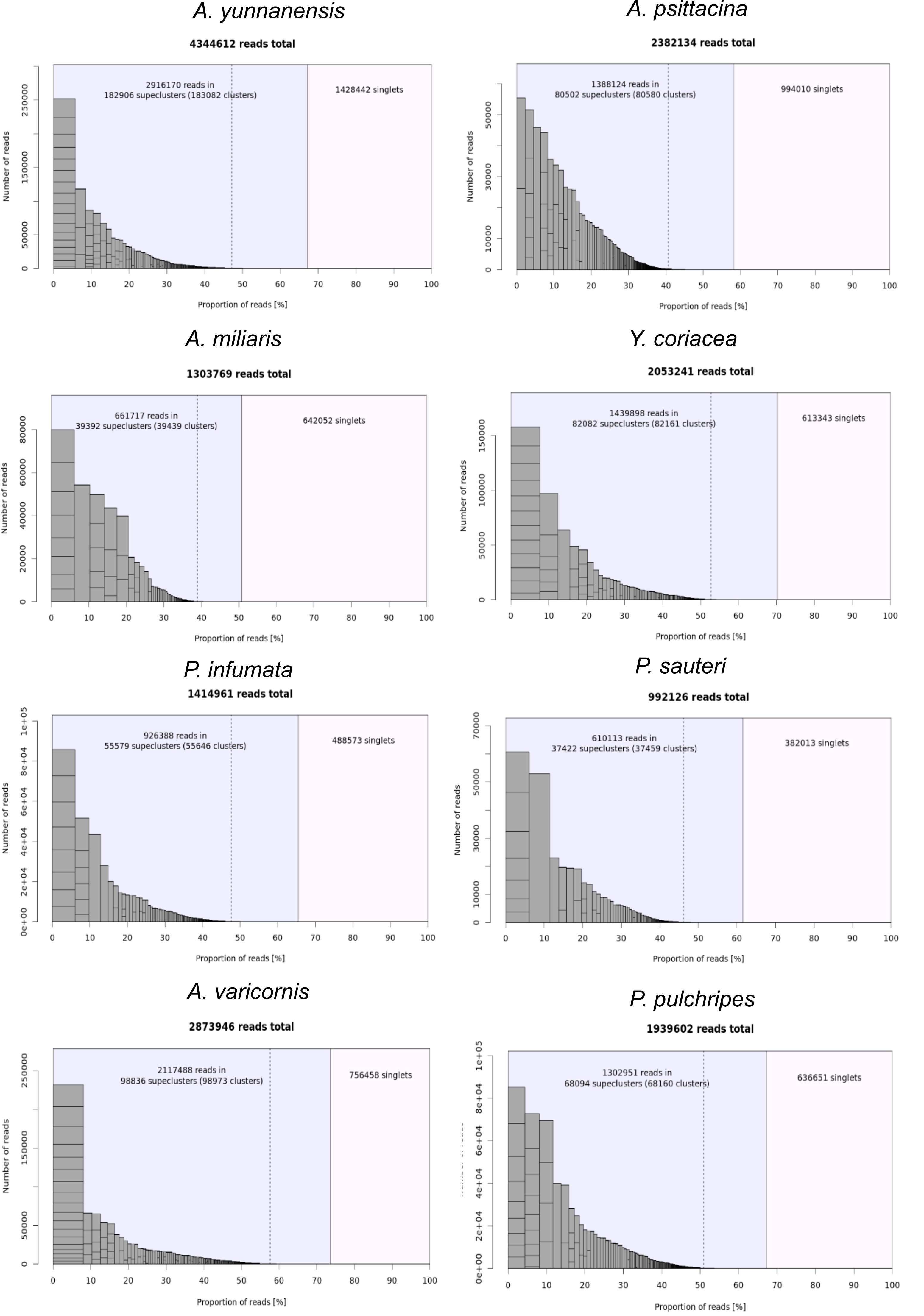

A. yunnanensis
4344612 reads total
2916170 reads in
182906 supeclusters (183082 clusters)
1428442 singlets
Number of reads
Proportion of reads [%]
A. psittacina
2382134 reads total
1388124 reads in
80502 supeclusters (80580 clusters)
994010 singlets
Number of reads
Proportion of reads [%]
A. miliaris
1303769 reads total
661717 reads in
39392 supeclusters (39439 clusters)
642052 singlets
Number of reads
Proportion of reads [%]
Y. coriacea
2053241 reads total
1439898 reads in
82082 supeclusters (82161 clusters)
613343 singlets
Number of reads
Proportion of reads [%]
P. infumata
1414961 reads total
926388 reads in
55579 supeclusters (55646 clusters)
488573 singlets
Number of reads
Proportion of reads [%]
P. sauteri
992126 reads total
610113 reads in
37422 supeclusters (37459 clusters)
382013 singlets
Number of reads
Proportion of reads [%]
A. varicornis
2873946 reads total
2117488 reads in
98836 supeclusters (98973 clusters)
756458 singlets
Number of reads
Proportion of reads [%]
P. pulchripes
1939602 reads total
1302951 reads in
68094 supeclusters (68160 clusters)
636651 singlets
Proportion of reads [%]

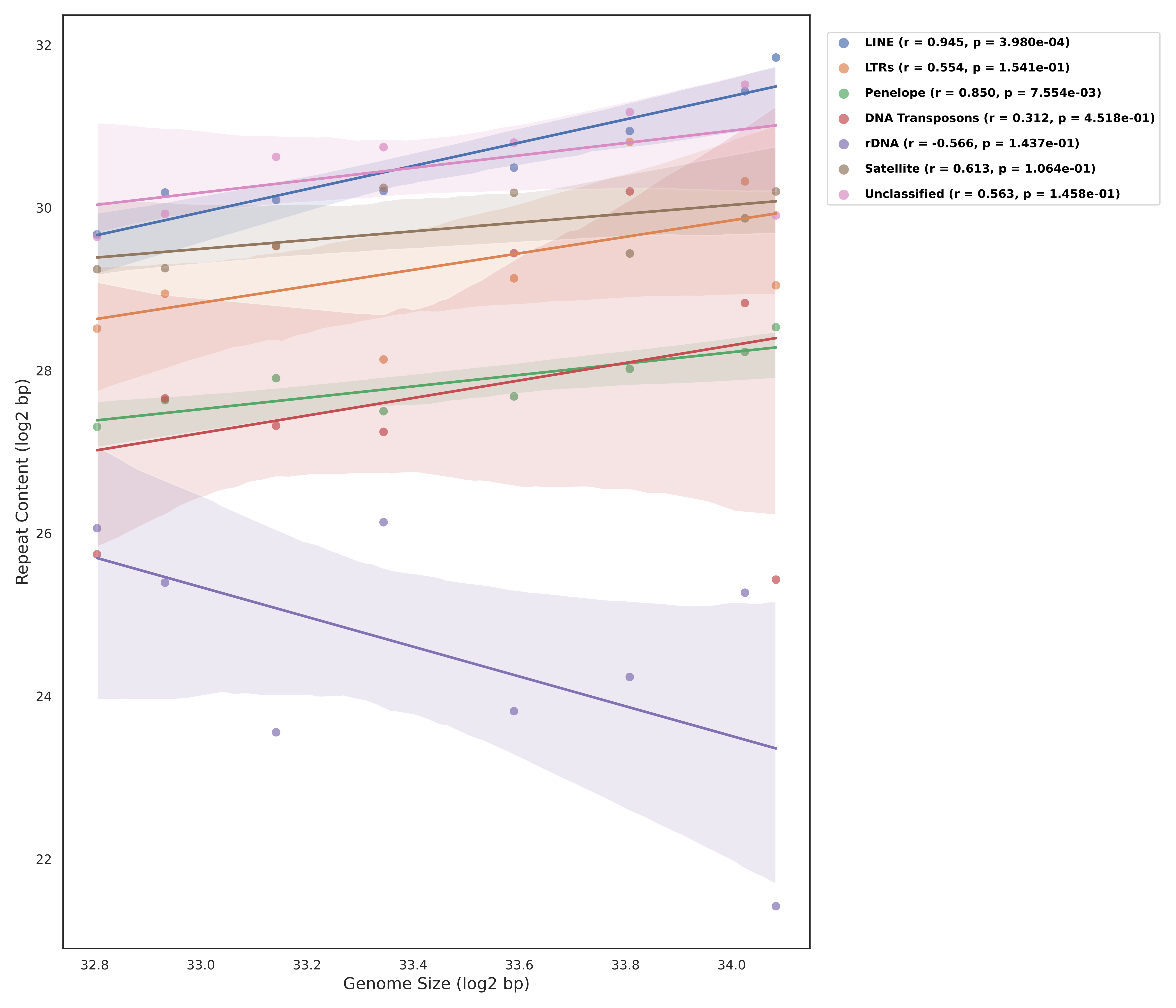
LINE (r = 0.945, p = 3.980e-04)
LTRs (r = 0.554, p = 1.541e-01)
Penelope (r = 0.850, p = 7.554e-03)
DNA Transposons (r = 0.312, p = 4.518e-01)
rDNA (r = -0.566, p = 1.437e-01)
Satellite (r = 0.613, p = 1.064e-01)
Unclassified (r = 0.563, p = 1.458e-01)
Repeat Content (log2 bp)
Genome Size (log2 bp)
32
30
28
26
24
22
32.8
33.0
33.2
33.4
33.6
33.8
34.0

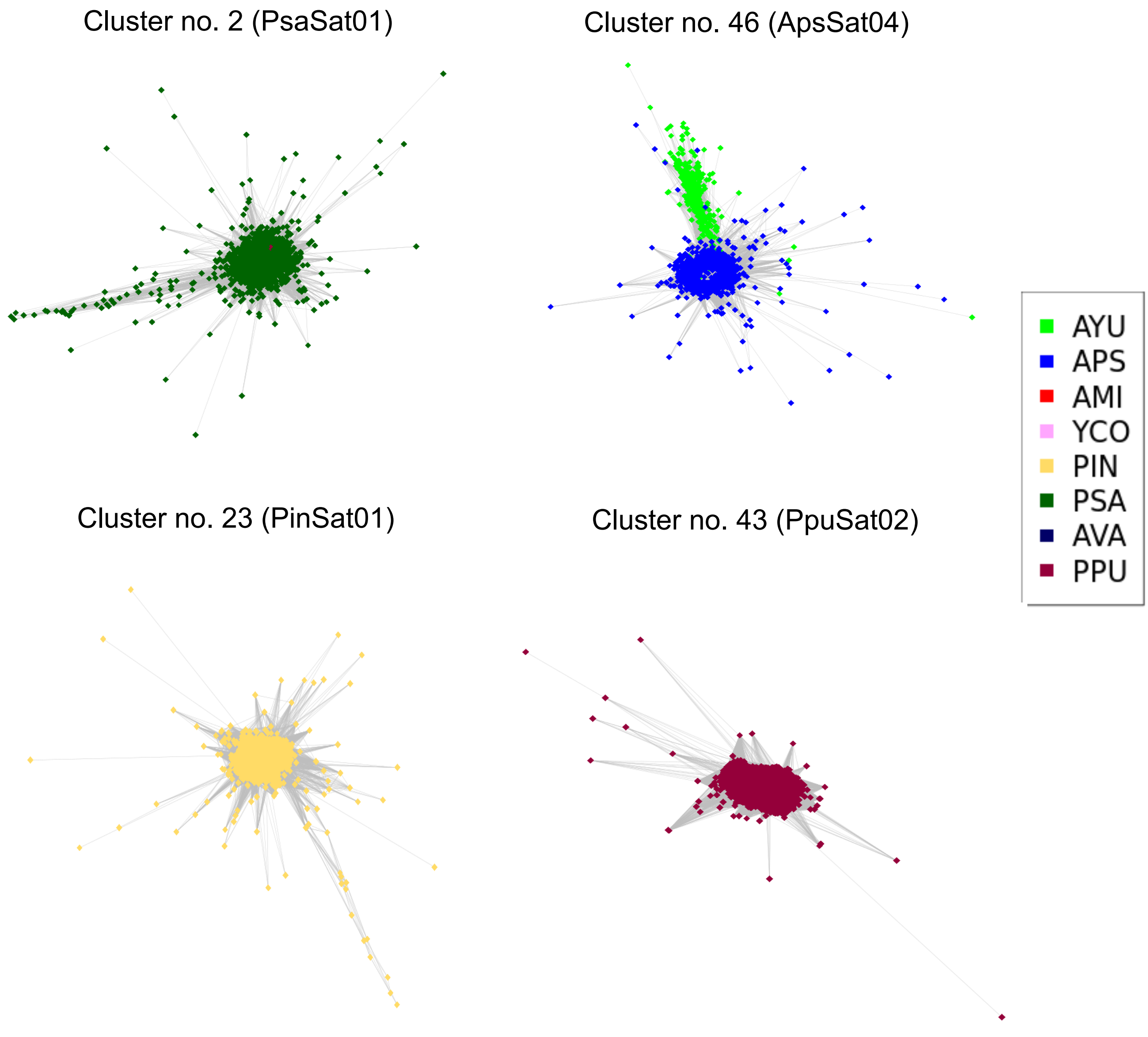

Cluster no. 2 (PsaSat01)
Cluster no. 46 (ApsSat04)
Cluster no. 23 (PinSat01)
Cluster no. 43 (PpuSat02)
AYU
APS
AMI
YCO
PIN
PSA
AVA
PPU

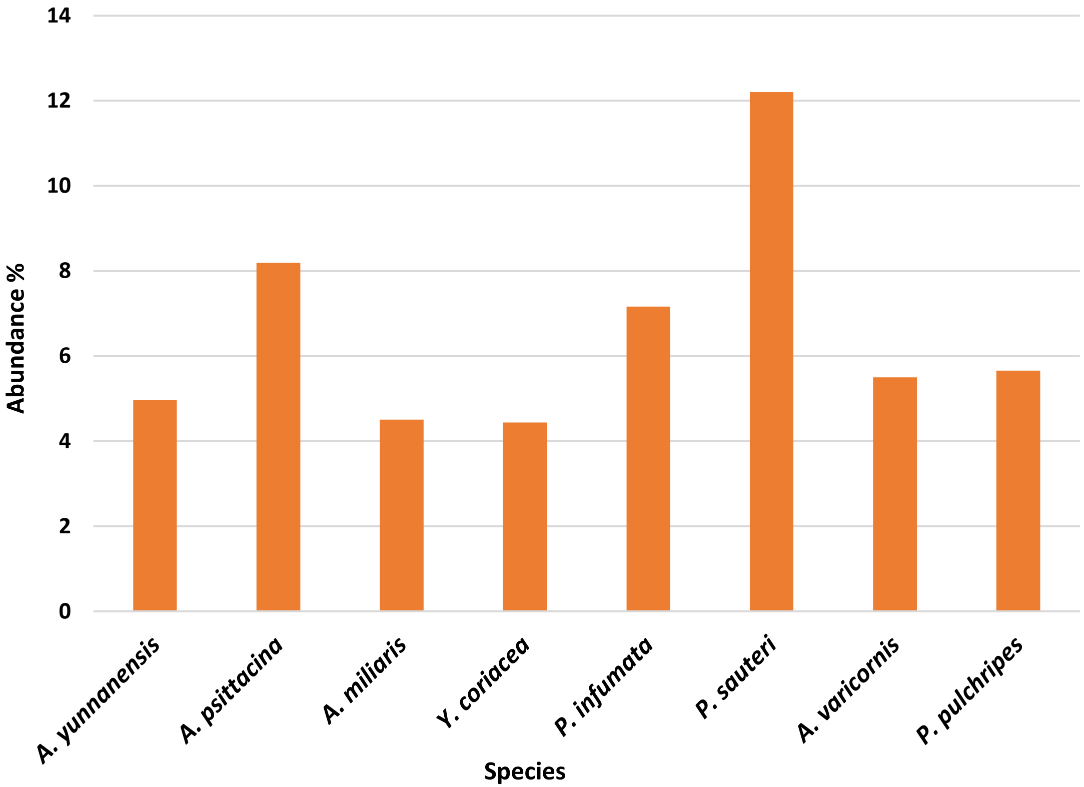

14
12
10
8
6
4
2
0
Abundance %
A. yunnanensis
A. psittacina
A. miliaris
Y. coriacea
P. infumata
P. sauteri
A. varicornis
P. pulchripes
Species

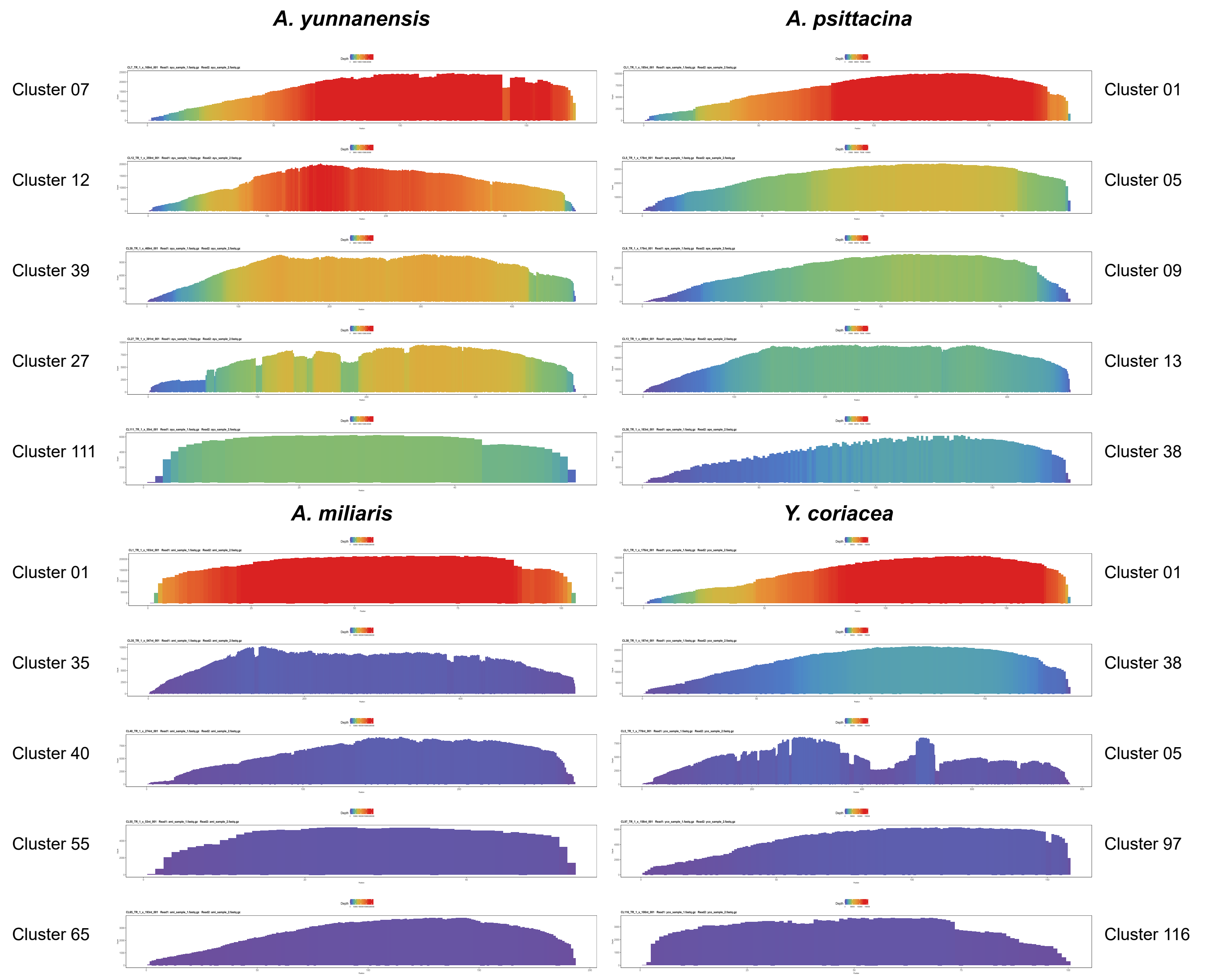
A. yunnanensis
A. psittacina
Cluster 07
Cluster 01
Cluster 12
Cluster 05
Cluster 39
Cluster 09
Cluster 27
Cluster 13
Cluster 111
Cluster 38
A. miliaris
Y. coriacea
Cluster 01
Cluster 01
Cluster 35
Cluster 38
Cluster 40
Cluster 05
Cluster 55
Cluster 97
Cluster 65
Cluster 116

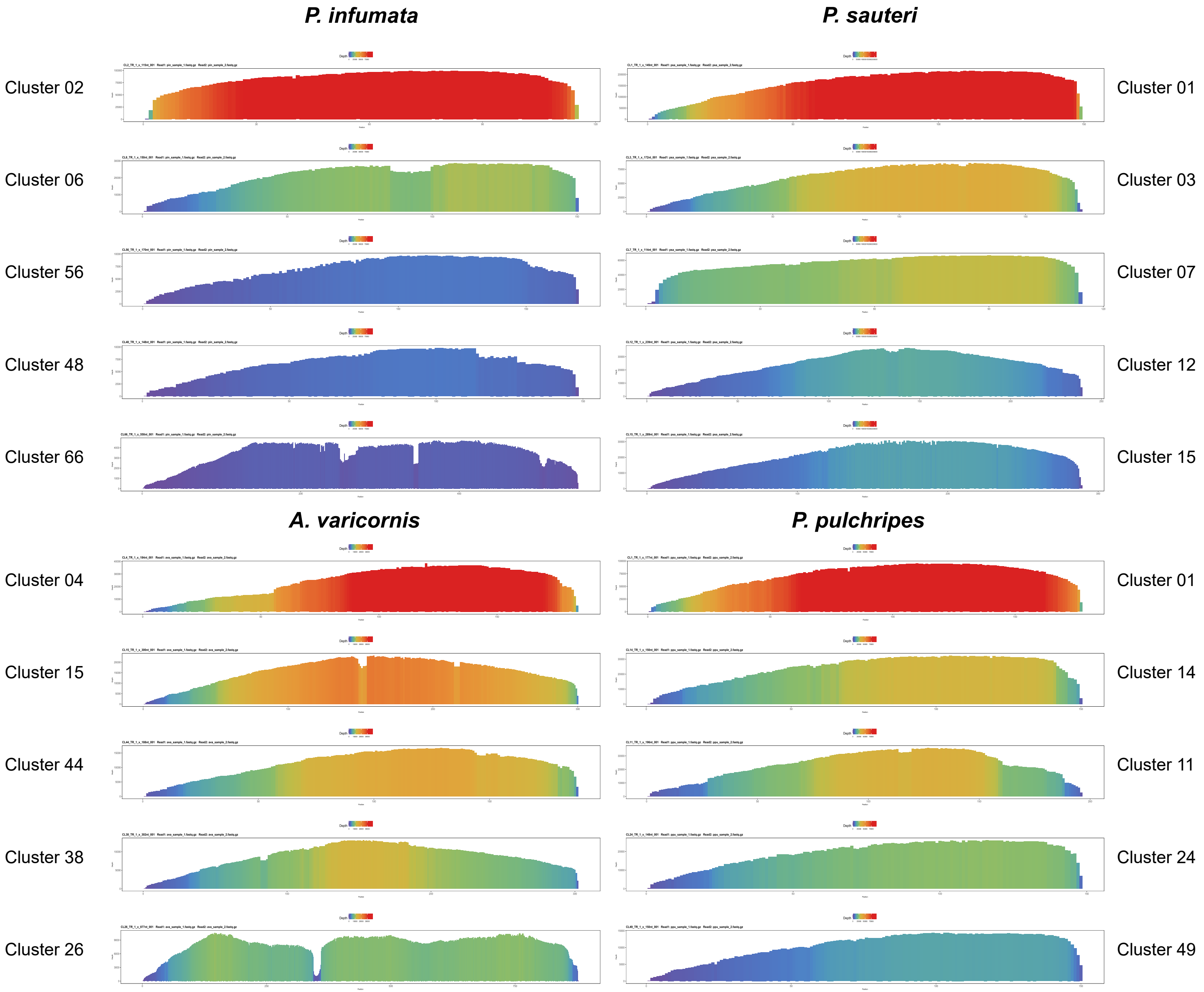

P. infumata
P. sauteri
Cluster 02
Cluster 01
Cluster 06
Cluster 03
Cluster 56
Cluster 07
Cluster 48
Cluster 12
Cluster 66
Cluster 15
A. varicornis
P. pulchripes
Cluster 04
Cluster 01
Cluster 15
Cluster 14
Cluster 44
Cluster 11
Cluster 38
Cluster 24
Cluster 26
Cluster 49
